# The *SlDUF789* Gene Family in Tomato: Genome-Wide Identification and Responsiveness to Hormonal and Abiotic Stress Signals

**DOI:** 10.3390/ijms27188014

**Published:** 2026-09-09

**Authors:** Wenbing Zou, Xinfang Chen, Zhipeng Wang, Weibiao Liao

**Affiliations:** College of Horticulture, Gansu Agricultural University, Lanzhou 730070, China; 1073325120768@st.gsau.edu.cn (W.Z.); 1073325010154@st.gsau.edu.cn (X.C.); 1073325120802@st.gsau.edu.cn (Z.W.)

**Keywords:** abiotic stress, bioinformatics analysis, dynamic expression analysis, hormone response, *SlDUF789* gene family, tomato

## Abstract

The DUF789 protein family comprises conserved domain proteins in plants, most of which remain functionally uncharacterized despite their involvement in growth, development, and stress responses. Moreover, systematic identification of the DUF789 family in tomato (*Solanum lycopersicum* L.) has not been reported. In this study, 11 *SlDUF789* members were identified from the tomato genome, unevenly distributed across seven chromosomes, with members within the same subgroup sharing similar exon–intron structures and conserved motifs. Promoter analysis revealed the enrichment of *cis*-acting elements associated with light, hormone, and stress responses. Quantitative real-time PCR (qRT-PCR) analysis revealed diverse expression patterns across tissues: most *SlDUF789* members exhibited relatively high expression levels in reproductive tissues, whereas *SlDUF789-7* was broadly expressed across all tissues, with elevated expression in fully open flowers and red-ripe fruits. Under stress and hormone treatments, *SlDUF789-7* exhibited the highest induction under ABA and salt stress; *SlDUF789-8* under MeJA and PEG; and *SlDUF789-11* under SA and low-light. Notably, *SlDUF789-7* responded strongly across all treatments, whereas *SlDUF789-11* showed sustained upregulation under low-light stress. This study provides the first systematic characterization of the *SlDUF789* gene family in tomato and establishes a foundation for future research on their biological functions and potential roles in stress responses.

## 1. Introduction

DUF789 is a plant-specific conserved domain family whose biochemical function remains uncharacterized; recent studies reveal that its members exhibit species-specific expansion, divergent protein lengths, and promoter enrichments in hormone- and stress-related elements, suggesting potential roles in plant stress responses. In the Pfam database, DUF families are typically named using the format “DUF” followed by a number; their common characteristic is a relatively conserved amino acid sequence, but they lack clear experimental functional annotations [1,2]. With the continuous advancement of plant genome sequencing and protein domain annotation, a growing number of DUF gene families have been uncovered. Although DUF proteins initially received little attention due to their unknown functions, a growing body of research indicates that they are not merely genomic annotation entries. Instead, they are associated with processes such as plant growth and development, organ formation, and environmental adaptation [3].

During plant growth and development, members of various DUF families have been shown to have well-defined biological functions. For example, several members of the DUF231/TBL family in *Arabidopsis* are involved in xylan acetylation and cell wall structure formation, indicating that DUF proteins can participate in cell wall polysaccharide modification processes [4]. Members of the DUF579 family have also been reported to be associated with cell wall polysaccharide metabolism, further supporting the role of DUF proteins in cell wall formation [5]. In addition, some DUF proteins are involved in chloroplast development, root development, and floral organ formation. For example, the DUF640 protein localizes to chloroplasts and participates in light-regulated seedling development [6]; members of the DUF828 and DUF966 families are involved in root development and tissue differentiation [7,8]; and DUF761 has been reported to participate in floral organ development [9]. These studies indicate that DUF proteins are not merely “unknown-function” genes, but may represent a class of functional proteins within plant developmental regulatory networks that have yet to be fully explored.

In recent years, DUF proteins have received widespread attention for their potential roles in stress responses. In *Arabidopsis*, *AtRDUF1*, which contains a DUF1117 domain, positively regulates the salt stress response; meanwhile, silencing both *AtRDUF1* and *AtRDUF2* reduces ABA-mediated drought tolerance [10]. *ESK1*, a member of the DUF231 family, is considered a negative regulator in the cold acclimation process, and its functional loss can affect the plant’s response to low temperatures [11]. In rice, DUF1644 family genes such as *OsSIDP366* and *OsSIDP361* can enhance the plant’s tolerance to drought and salt stress [12,13]; overexpression of *OsDUF946* also enhances rice resistance to high-salinity and drought stress [14]. In wheat, *TaDUF966-9B* has been shown to be involved in the salt stress response, and *TaSRHP*, a DUF581 family gene, enhances *Arabidopsis* tolerance to salt and drought stress when overexpressed [15,16]. Similarly, transgenic cotton overexpressing *AmDUF1517* exhibited enhanced tolerance to salt, drought, and cold stress [17]. In sweet potato, *IbDUF668-6*, *-7*, *-11*, and *-13* are significantly upregulated under ABA, drought, and NaCl treatments [18]; in ginger, some *ZoDUF668* genes exhibited induced expression under low-temperature and waterlogging stress [19]. Thus, DUF genes not only participate in plant growth and development, but may also act as conditionally induced genes in responses to hormones and abiotic stresses, which may contribute to plants’ adaptation to complex environments.

Tomato (*Solanum lycopersicum* L.) is an important horticultural crop widely cultivated worldwide and serves as a key model organism for studying fleshy fruit development, Solanaceae genomics, and stress physiology [20,21]. During protected and open-field cultivation, adverse environmental conditions such as salt stress, drought-related osmotic stress, and low-light levels often limit tomato plant growth, photosynthetic performance, flowering and fruit set, yield formation, and fruit quality [22,23,24]. Genome-wide identification of the DUF789 family in *Arabidopsis*, cotton, and soybean has revealed stress-associated promoter elements and induced expression under abiotic stresses, suggesting its involvement in stress responses [25,26,27]. However, the tomato DUF789 gene family has not yet been systematically characterized. To fill this knowledge gap, this study identified 11 *SlDUF789* members from the tomato genome and conducted a systematic analysis of their chromosomal distribution, the physicochemical properties of the proteins, gene structure, conserved motifs, phylogenetic relationships, collinearity, and promoter characteristics. Furthermore, by integrating expression profiles across different tissues with qRT-PCR expression analyses under treatments with ABA, MeJA, SA, NaCl, PEG, and low-light, this study provides a systematic characterization of the evolutionary and expression characteristics of the DUF789 gene family in tomato, offering a basis for future functional validation of their potential roles in plant development and abiotic stress responses.

## 2. Results

### 2.1. Genome-Wide Identification and Chromosomal Localization of the SlDUF789 Gene Family

**Figure 1:**
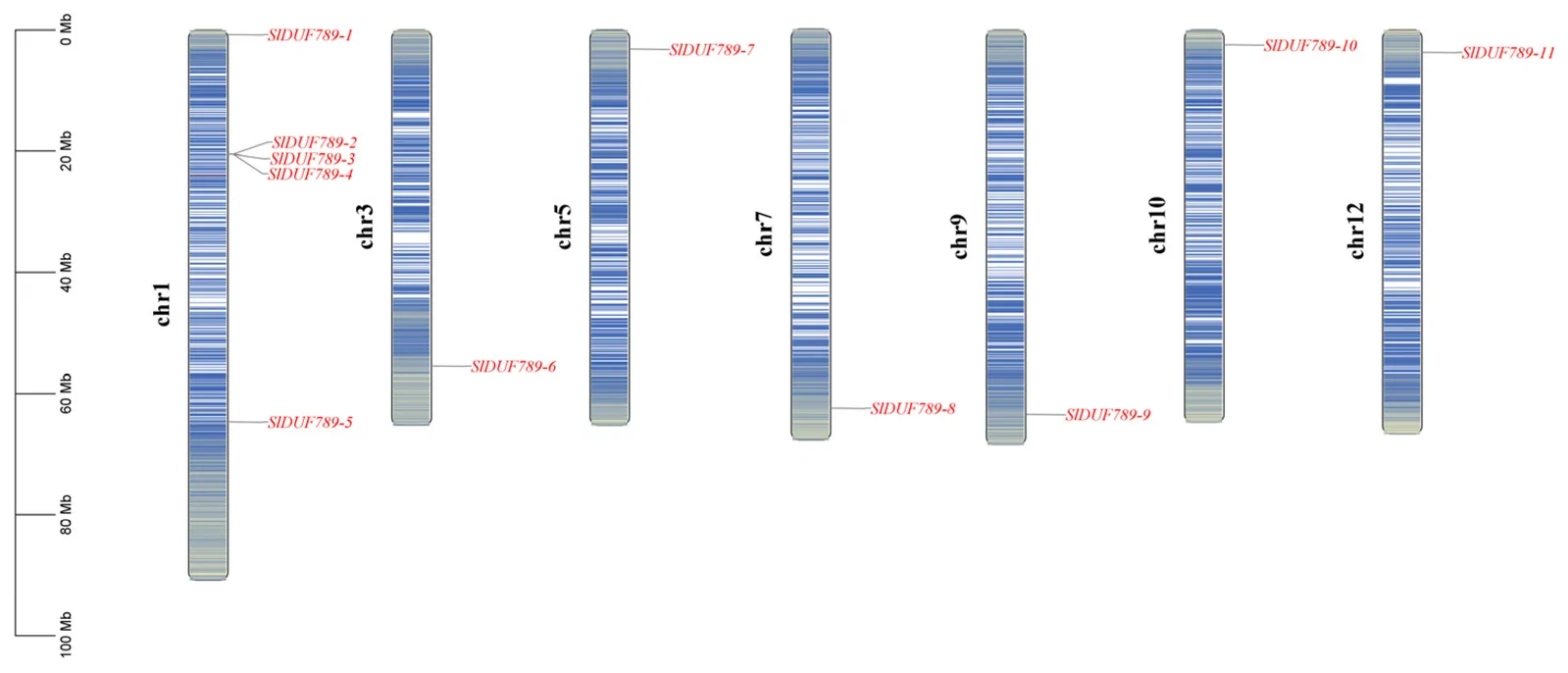
Chromosomal localization of the *SlDUF789* gene family. Vertical bars represent the 11 tomato chromosomes, arranged in ascending order of chromosome number (chr1, chr3, chr5, chr7, chr9, chr10, and chr12). The horizontal axis indicates the physical length of each chromosome in megabases (Mb). Chromosome numbers are shown in black, and gene identifiers (*SlDUF789-1* to *SlDUF789-11*) are highlighted in red.

To identify members of the tomato DUF789 family, we used the DUF789 domain HMM profile from Pfam to screen the tomato reference genome (ITAG 4.0) with TBtools (v2.0) software. The analysis identified a total of 11 non-redundant *SlDUF789* genes. Based on their physical locations on the chromosomes, we named them sequentially *SlDUF789-1* (*Solyc01g006080*) through *SlDUF789-11* (*Solyc12g010730*). Chromosomal localization analysis revealed that these 11 *SlDUF789* genes are unevenly distributed across chromosomes 1, 3, 5, 7, 9, 10, and 12. Chromosome 1 contains the majority of this family’s members (5/11), indicating a local amplification in that region; the remaining chromosomes each contain only one member. This uneven distribution suggests that during the evolution of the tomato, the *SlDUF789* family may have undergone chromosome-specific duplication and retention events (Figure 1).

The SlDUF789 proteins exhibit significant heterogeneity in their predicted physicochemical properties. Their amino acid lengths range from 87 to 1097 amino acids, and their molecular weights range from 10.03 to 123.47 kDa, indicating significant differences in protein size among family members. Notably, SlDUF789-6 and SlDUF789-9 have theoretical isoelectric points greater than 7.0, while the remaining members are acidic (pI < 7.0), suggesting that the family as a whole carries a net negative charge under physiological pH conditions. The instability indices of SlDUF789-5 through SlDUF789-11 are all greater than 40, indicating that these proteins may be unstable. Furthermore, the grand average of hydropathicity values for all members was negative, indicating that SlDUF789 family proteins are generally highly hydrophilic. Subcellular localization predictions suggest that SlDUF789 proteins may be distributed across multiple cellular structures, including the nucleus, plasma membrane, cell wall, and chloroplasts, implying that family members may participate in various biological processes by localizing to different functional sites (Table 1).

### 2.2. Prediction and Analysis of the Secondary and Tertiary Structures of the SlDUF789 Proteins

**Figure 2:**
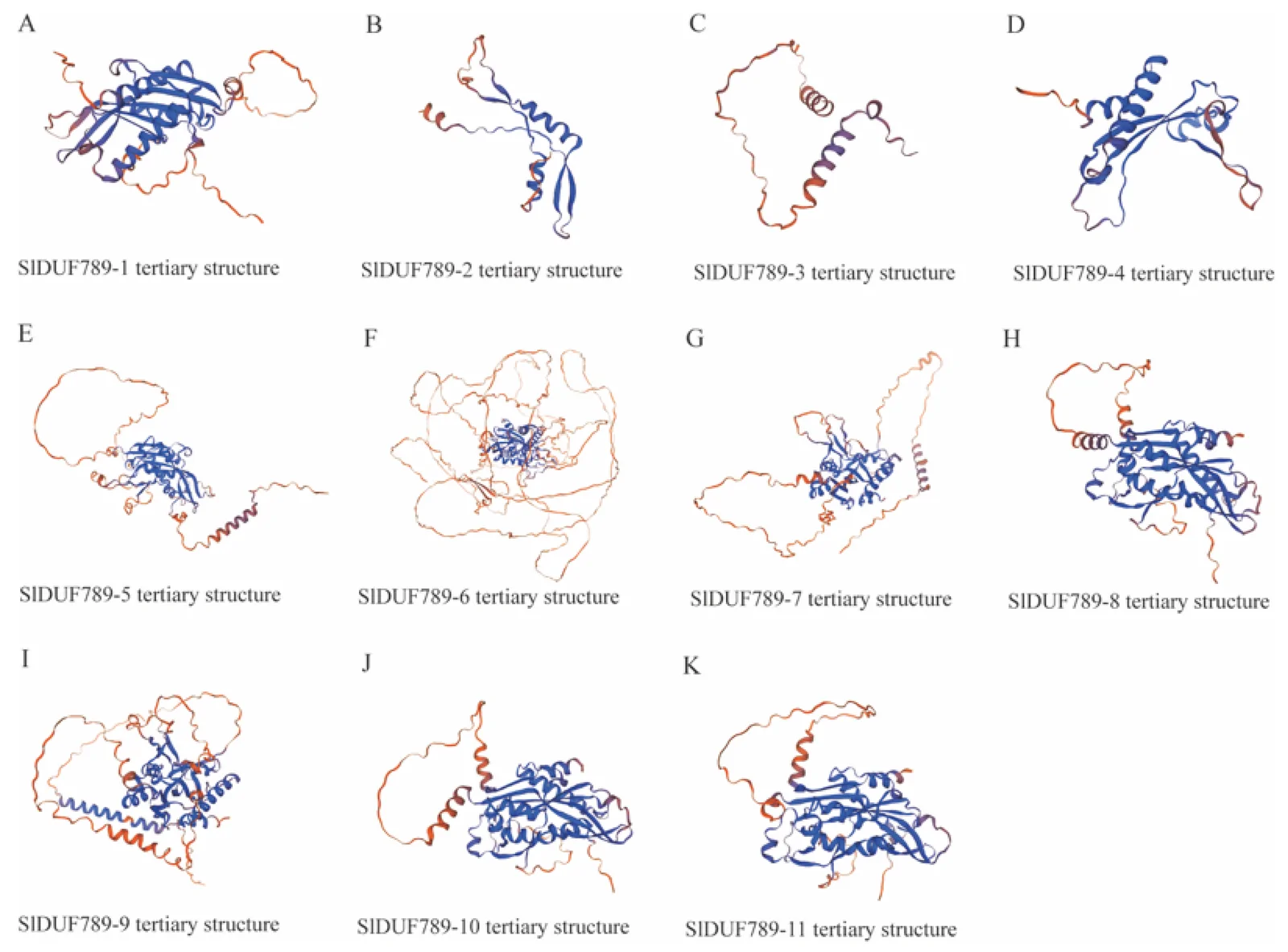
Predicted tertiary structures of SlDUF789 proteins. (**A**–**K**) Structures of SlDUF789-1 to SlDUF789-11, respectively. Color scheme: blue, α-helices; purple, extended strands; red, random coils.

To evaluate the structural characteristics of the SlDUF789 proteins, their secondary structures were predicted. The results show that the α-helix content ranges from 12.76% to 30.10%, the extended strand content ranges from 6.02% to 12.64%, the random coil content ranges from 61.05% to 81.22%, and the β-turn content is predicted to be 0% (Table 2). These results indicate that the SlDUF789 proteins consist primarily of random coils, α-helices, and extended chains, suggesting that they possess high structural flexibility. Tertiary structure predictions further reveal differences in the overall three-dimensional conformations of SlDUF789 family members, which are particularly evident in proteins with significant variations in length or structural complexity (Figure 2). Therefore, the predicted secondary and tertiary structures not only support structural conservation within the SlDUF789 family, but also reflect its structural diversity.

### 2.3. Analysis of Conserved Motifs and Gene Structures in the SlDUF789 Family

**Figure 3:**
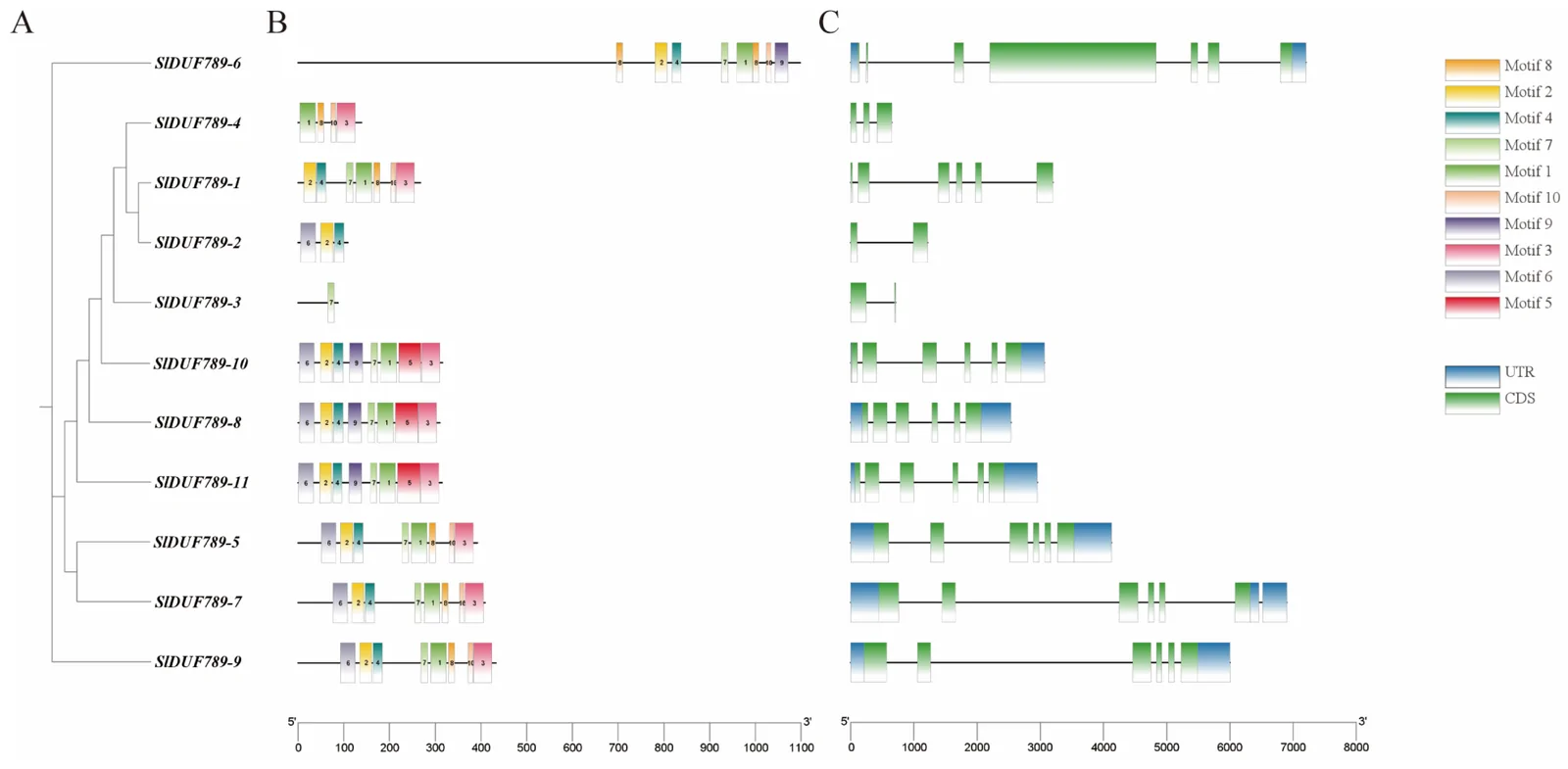
Phylogenetic relationship, conserved protein motifs, and gene structure of the *SlDUF789* gene family. (**A**) Phylogenetic analysis. A maximum likelihood phylogenetic tree was constructed using MEGA11 software based on the amino acid sequences of 11 SlDUF789 proteins. (**B**) Composition of conserved motifs. The conserved motifs of 11 SlDUF789 proteins were identified using the MEME program. Motifs 1 to 10 are represented by differently colored boxes. The scale bar below indicates the length of the amino acid sequence. (**C**) Gene structure analysis. The exon–intron structure of 11 *SlDUF789* genes. Green boxes represent the coding sequence (CDS), blue boxes represent the non-translated region (UTR), and black lines represent introns. The scale bar below indicates the lengths of exons, introns, and UTRs.

Phylogenetic analysis classified the 11 *SlDUF789* members into distinct clades (Figure 3A). Based on this, we used the online MEME tool to identify 10 conserved motifs in the 11 SlDUF789 proteins (Figure 3B), and simultaneously visualized the exon–intron structures of these genes using TBtools software (Figure 3C). Examination of conserved motifs and gene structures showed that members of the *SlDUF789* family exhibit both structural conservation and significant diversity. The number of non-coding regions ranges from 1 to 6, while the number of coding regions ranges from 2 to 7. Specifically, *SlDUF789-1*, *-5*, *-8*, *-9*, *-10*, and *-11* contain five introns and six exons; *SlDUF789-6* and *SlDUF789-7* contain six introns and seven exons; and the remaining members contain fewer. Phylogenetically closely related members generally share similar motif arrangements and exon–intron structures. For example, the clades containing *SlDUF789-8*, *-10*, and *-11*, as well as those containing *SlDUF789-5*, *-7*, and *-9*, exhibit similar motif arrangements and gene structures, suggesting that these members may share a common evolutionary origin and potential functions. In contrast, *SlDUF789-3* and *SlDUF789-6* show marked differences in motif number, protein length, and gene structure, indicating that they may have undergone structural variation during evolution and functional divergence.

### 2.4. Construction of a Phylogenetic Tree for the DUF789 Proteins

**Figure 4:**
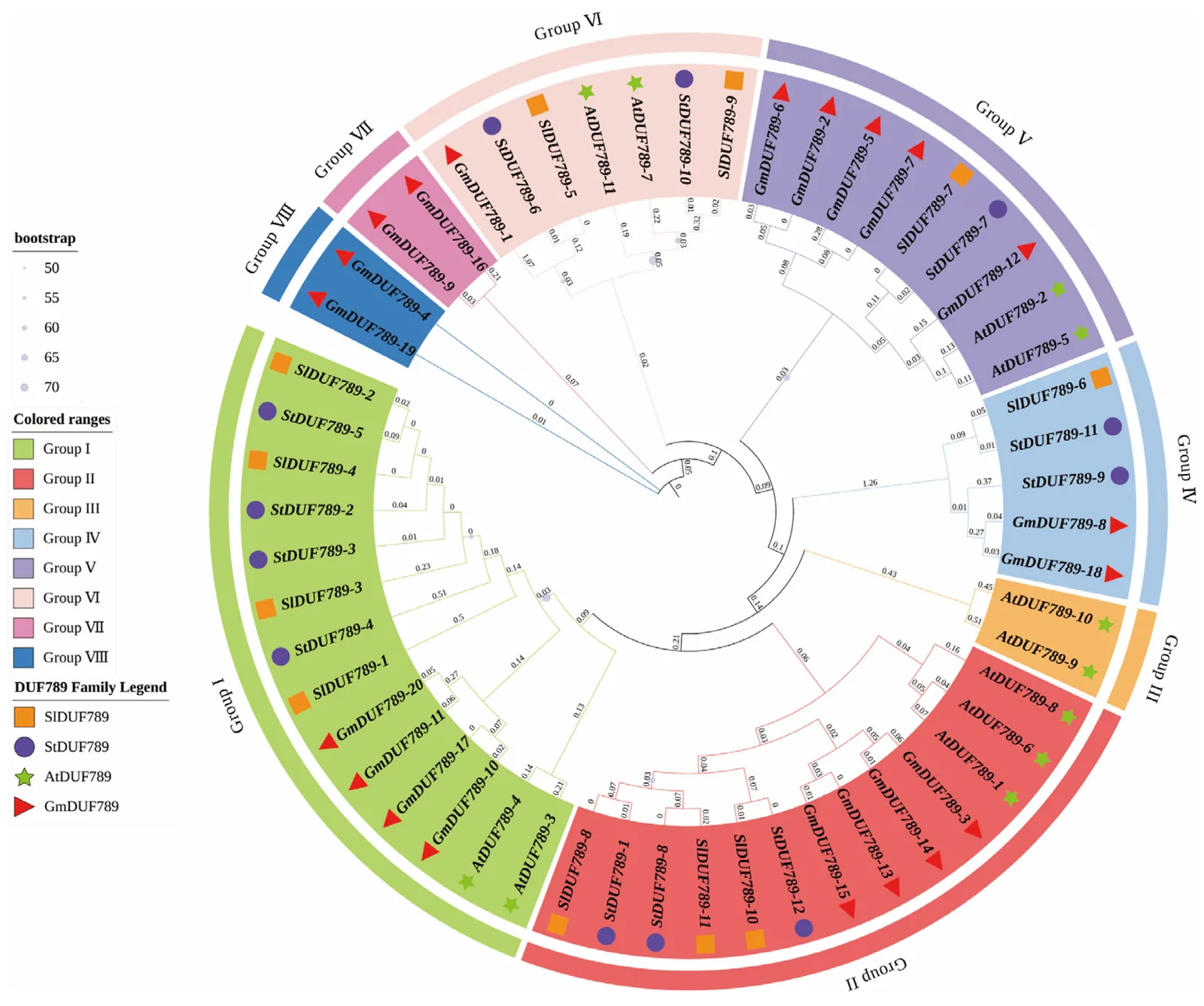
Phylogenetic relationships among DUF789 proteins from four species—tomato, potato, *Arabidopsis*, and soybean—were inferred using an ML tree generated in MEGA11 with amino acid sequences. The resulting phylogeny was resolved into eight distinct clades (Groups I–VIII), color-coded for clarity. Bootstrap values are indicated at the branch nodes. Different shapes and colors of labels represent different species: orange square for tomato (SlDUF789), purple circle for potato (StDUF789), green star for *Arabidopsis thaliana* (AtDUF789), and red triangle for soybean (GmDUF789).

To examine evolutionary relationships, we constructed a phylogenetic tree using 11 SlDUF789, 11 AtDUF789, 12 StDUF789, and 20 GmDUF789 protein sequences (Figure 4). The results showed that based on homology, members of the DUF789 family can be classified into eight subgroups, five of which (Groups I, II, IV, V, and VI) contain SlDUF789 members, indicating that this family exhibits both evolutionary conservation and functional diversification in tomato. *SlDUF789-1*, *-2*, *-3*, and *-4* are predominantly distributed in Group I and may represent a relatively conserved class of members within the tomato DUF789 family; *SlDUF789-8*, *-10*, and *-11* are located in Group II and may share a similar evolutionary origin and potential functional redundancy; whereas *SlDUF789-5*, *-6*, *-7*, and *-9* are distributed across different branches, suggesting that they may have undergone functional diversification during evolution.

### 2.5. Collinearity Analysis of the Tomato DUF789 Gene Family

**Figure 5:**
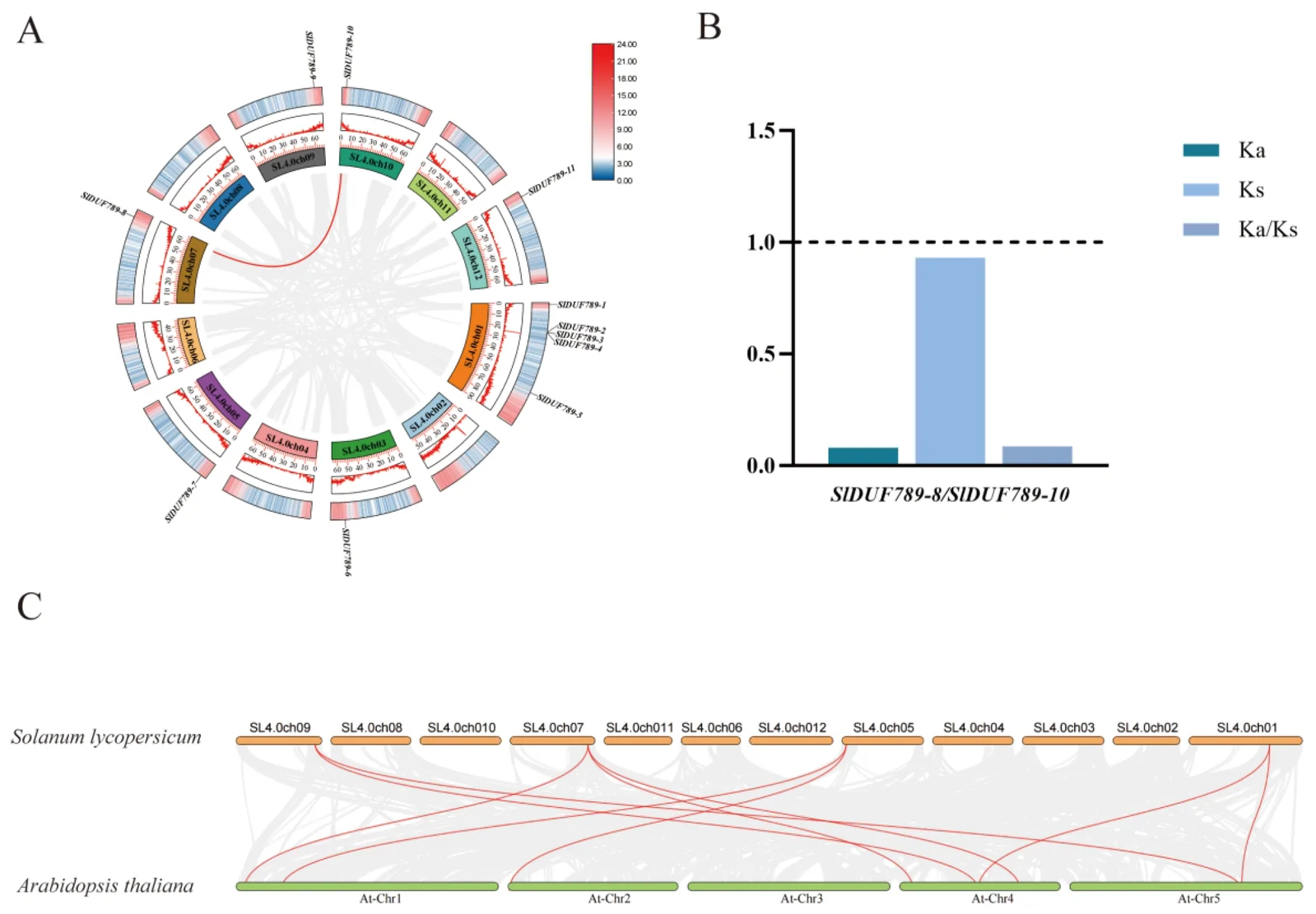
Intraspecific synteny, selective pressure, and interspecific collinearity analyses of the tomato *DUF789* gene family. (**A**) Circular intraspecific collinearity map of *SlDUF789* genes, illustrating genomic duplication events among homologs across tomato chromosomes (SL4.0ch01–SL4.0ch12). (**B**) Selective pressure analysis of paralogous *SlDUF789* gene pairs (from A). Ka, Ks, and *Ka/Ks* ratios are shown; the dashed line at *Ka/Ks* = 1.0 indicates purifying selection (*Ka/Ks* < 1). (**C**) Interspecific collinearity between tomato (*Solanum lycopersicum*) and *Arabidopsis thaliana*. Red lines connect homologous *DUF789* gene pairs between tomato chromosomes (SL4.0ch01–SL4.0ch12) and A. thaliana chromosomes (AtChr1–AtChr5).

Intraspecific collinearity analysis of tomato revealed that *SlDUF789-8* and *SlDUF789-10* are the only collinear gene pair detected in the DUF789 family, suggesting that these two genes may have originated from a segmental duplication event (Figure 5A). The *Ka/Ks* ratio is below 1 (Figure 5B), indicating that this gene pair has been subject to purifying selection during evolution, suggesting that *SlDUF789-8* and *SlDUF789-10* may exhibit functional redundancy or similar biological functions. Furthermore, an interspecific collinearity analysis between tomato and *Arabidopsis* revealed nine pairs of DUF789 homologous genes between these two species (Figure 5C), indicating that the DUF789 family exhibits a certain degree of evolutionary conservation in dicotyledons.

### 2.6. Analysis of Cis-Acting Elements in the Promoters of SlDUF789 Genes

**Figure 6:**
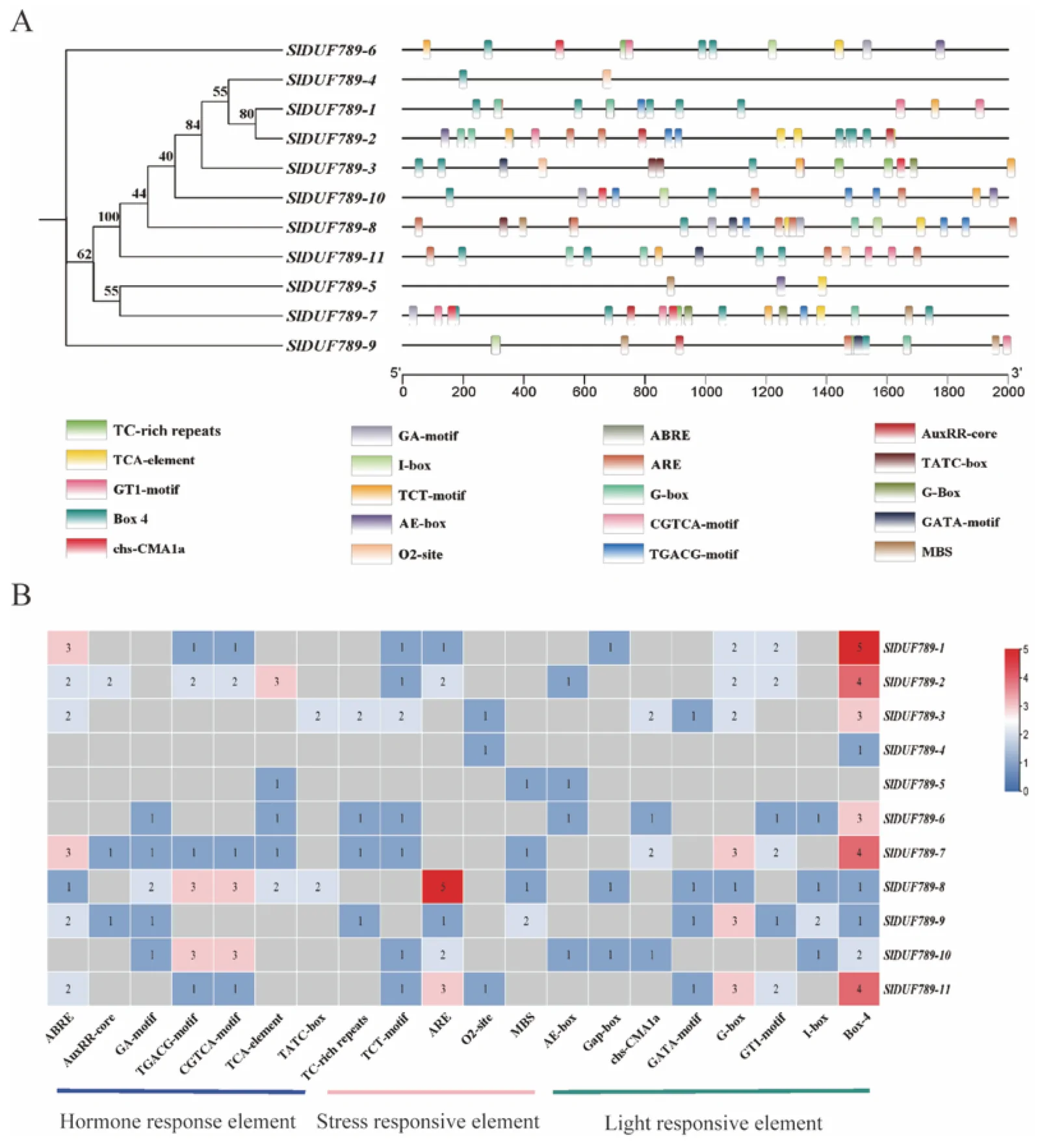
Identification and quantitative analysis of *cis*-regulatory elements in the promoter region of *SlDUF789* genes. (**A**) Distribution of *cis*-regulatory elements in 11 promoter regions of *SlDUF789* genes. The phylogenetic tree is shown on the left, and the corresponding *cis*-regulatory elements are indicated by colored boxes. (**B**) Quantitative analysis of *cis*-regulatory elements in *SlDUF789* genes. The heatmap shows the number of each *cis*-regulatory element in each gene (color intensity and value), and is divided into three categories: hormone-responsive (blue), stress-responsive (pink), and light-responsive (green).

To gain insight into *SlDUF789* functions, we analyzed the 2000 bp upstream regions of each family member using PlantCARE to identify potential *cis*-acting regulatory elements (Figure 6). The results show that *SlDUF789* promoters are enriched in three categories of *cis*-elements: light-responsive (42), hormone-responsive (34), and stress-responsive (24), with light-responsive elements being the most abundant. Light-responsive elements such as Box 4, G-box, GT1-motif, I-box, and GATA-motif are widely distributed in the promoters of all family members (except *SlDUF789-5*). Regarding hormone response, the promoters contain elements such as ABRE (ABA response) and TCA elements (SA response), as well as the TGACG/CGTCA motif (MeJA response). Furthermore, the presence of stress-related elements such as MBS (drought response), ARE (anaerobic response), and TC-rich repeat sequences (associated with defense) suggests a potential role of this family in abiotic stress responses. Notably, different members contain distinct potential binding sites: *SlDUF789-1* and *SlDUF789-7* are rich in ABREs, suggesting that they may be responsive to abscisic acid (ABA); *SlDUF789-8* and *SlDUF789-10* contain TGACG and CGTCA motifs, suggesting that they may be associated with methyl jasmonic acid (MeJA) responses; whereas *SlDUF789-2*, *-5*, *-6*, *-7*, and *-8* carry TCA elements, indicating that their expression may be influenced by salicylic acid (SA). These findings suggest that light signals may be an important environmental factor regulating the expression of the *SlDUF789* family, and that this family may also be regulated by various hormones and stress signals.

### 2.7. Tissue-Specific Expression Analysis of SlDUF789 Genes

**Figure 7:**
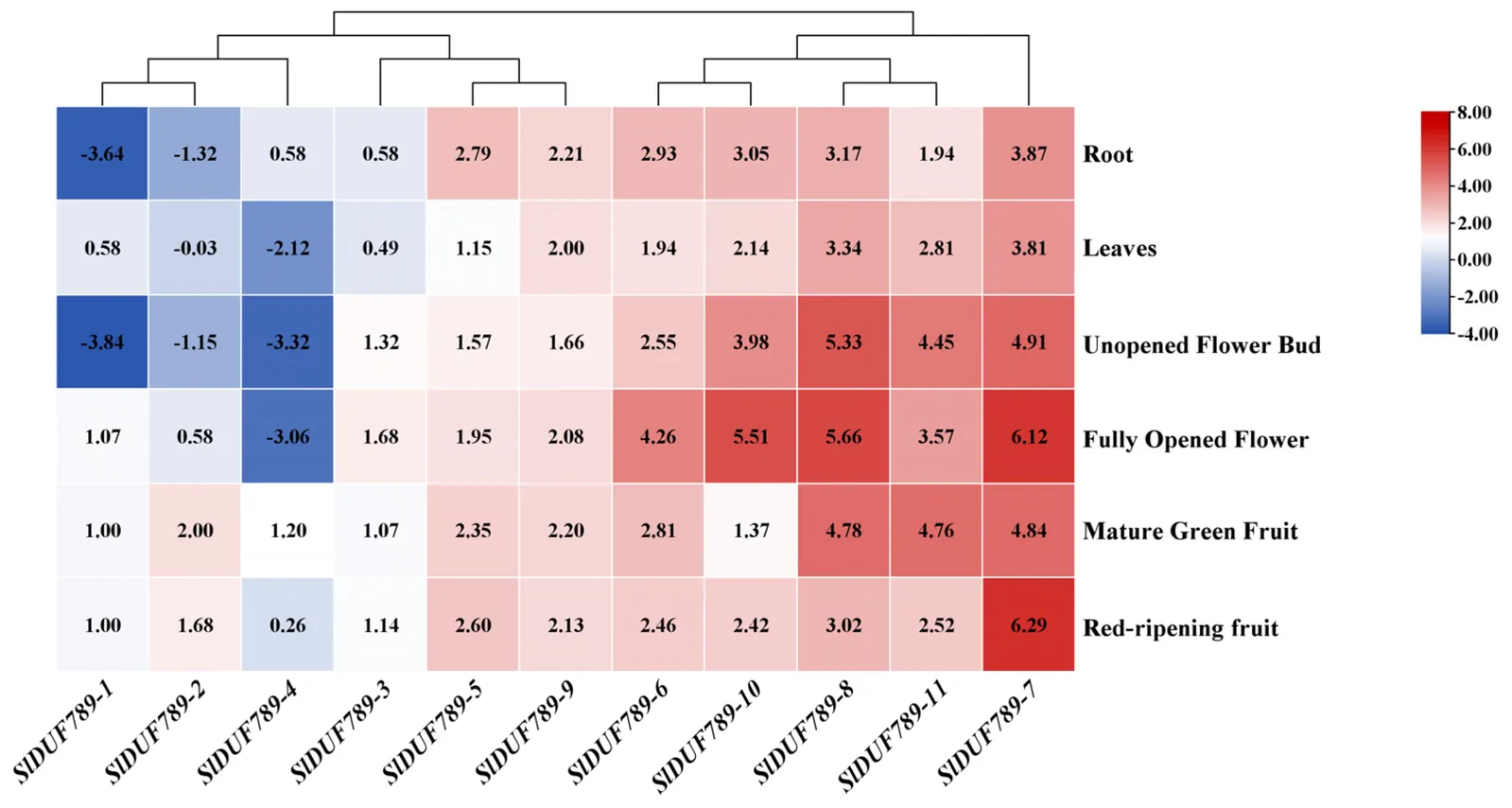
Expression profiles of *SlDUF789* genes across tomato tissues are displayed in a heatmap, constructed using log2-transformed fold change values. The color gradient from blue (low) through white (medium) to red (high) illustrates the tissue-specific expression patterns of the family members.

To characterize tissue-specific expression patterns of the *SlDUF789* gene family, six representative tissues—roots, leaves, unopened flower buds, fully opened flowers, mature-green fruits, and red-ripe fruits—were analyzed by qRT-PCR (Figure 7). The results showed that the expression patterns of family members varied significantly across different tissues. *SlDUF789-6*, *-8*, and *-10* reached peak expression levels in fully opened flowers, demonstrating distinct floral tissue specificity; *SlDUF789-11* was highly expressed in unopened flower buds and mature-green fruits, with the lowest expression levels in roots, suggesting that it may be involved in floral organ development and early fruit formation. Notably, *SlDUF789-7* maintained high transcriptional levels in all tested tissues and reached peak expression in fully opened flowers and red-ripe fruits, suggesting that it may be involved in floral organ development and fruit ripening. In contrast, *SlDUF789-1*, *-2*, *-3*, and *-4* exhibited low expression levels in all tested tissues, while *SlDUF789-5* and *SlDUF789-9* showed relatively high expression levels. These results indicate that members of the DUF789 family may be involved in tomato flower and fruit development, laying the foundation for subsequent functional validation.

### 2.8. Expression Patterns of SlDUF789 Genes Under Hormone Treatments

**Figure 8:**
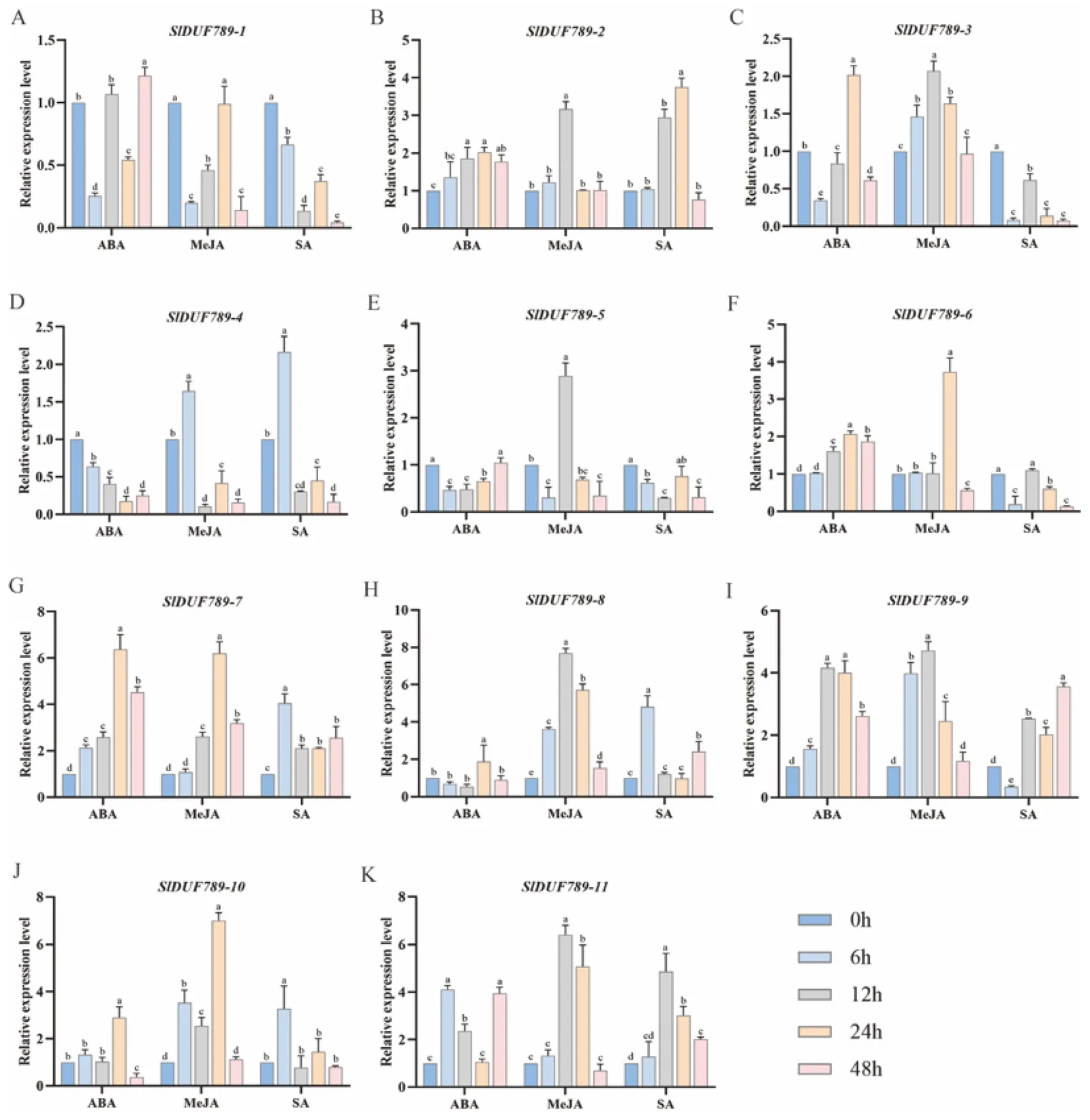
Dynamic expression patterns of *SlDUF789* genes under abscisic acid (ABA), methyl jasmonate (MeJA), and salicylic acid (SA) treatments (**A**–**K**). Panels (**A**–**K**) display the relative expression levels of *SlDUF789* family members at 0, 6, 12, 24, and 48 h post-treatment. Data are presented as mean ± SE (*n* = 3 biological replicates). Relative expression levels were calculated using the 2^^−ΔΔCt^ method and normalized to *SlActin*. Within the same hormone treatment, different lowercase letters denote significant differences among time points (*p* < 0.05, Duncan’s multiple range test).

To investigate the expression patterns of the *SlDUF789* genes under different hormonal treatments, this study analyzed the transcriptional levels of 11 *SlDUF789* genes at 0, 6, 12, 24, and 48 h following treatment with ABA, MeJA, and SA (Figure 8). Under ABA treatment, *SlDUF789-7* exhibited the strongest response to ABA, with expression levels rising continuously and peaking at 24 h, reaching 6.38 times that of the control. *SlDUF789-9* showed the highest expression at 12 h and maintained a relatively high expression level at 24 h, at 4.17 times and 4 times that of the control, respectively. *SlDUF789-10* reached its highest expression level at 24 h, which was 2.9 times that of the control; *SlDUF789-11* showed higher expression levels at 6 and 48 h, at 4.1 times and 3.94 times that of the control, respectively. In contrast, *SlDUF789-1*, *-4*, and *-5* exhibited lower expression levels or remained relatively stable throughout the treatment period.

Under MeJA treatment, a broader range of *SlDUF789* members were induced compared to ABA treatment. *SlDUF789-2*, *-3*, *-5*, *-8*, *-9*, and *-11* all reached their peak expression 12 h after treatment. Among these, *SlDUF789-8* was strongly induced under MeJA treatment, with an expression level 7.98 times higher than that of the control at the 12-h time point. *SlDUF789-6*, *-7*, and *-10* peaked at 24 h, with expression levels 3.74, 6.01, and 7.00 times higher than the control, respectively; among these, *SlDUF789-7* maintained a relatively high level even at 48 h.

Under SA treatment, *SlDUF789-7*, *-8*, and *-10* exhibited a rapid response to SA, reaching peak expression levels at 6 h—4.05, 4.82, and 3.27 times that of the control, respectively—suggesting that they may be involved in the early SA response. *SlDUF789-11*, *-2*, and *-9* reached their peaks at 12, 24, and 48 h, respectively, with expression levels 4.86-, 3.75-, and 3.56-fold higher than the control. In contrast, *SlDUF789-1*, *-3*, *-4*, *-5*, and *-6* showed weaker expression or were significantly downregulated under SA treatment; among these, *SlDUF789-1*, *-3*, and *-5* maintained expression levels below those of the control group throughout the entire treatment period.

### 2.9. Expression Patterns of the SlDUF789 Genes Under Abiotic Stress Conditions

**Figure 9:**
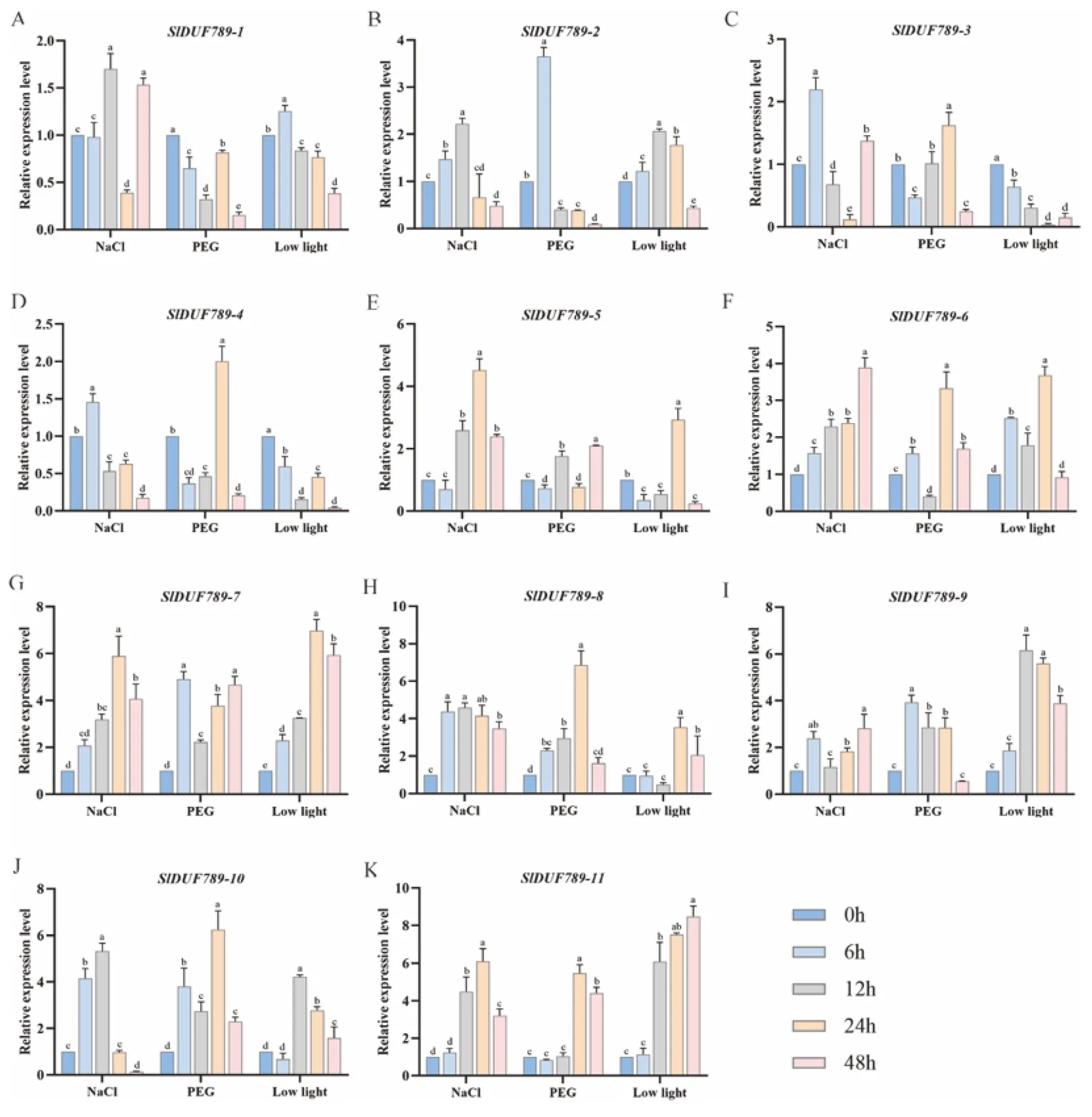
Dynamic expression patterns of *SlDUF789* genes under sodium chloride (NaCl), polyethylene glycol-6000 (PEG-6000), and low-light stress treatments (**A**–**K**). Panels (**A**–**K**) display the relative expression levels of *SlDUF789* family members at 0, 6, 12, 24, and 48 h post-treatment. Data are presented as mean ± SE (*n* = 3 biological replicates). Relative expression levels were calculated using the 2^^−ΔΔCt^ method and normalized to *SlActin*. Within the same stress treatment, different lowercase letters denote significant differences among time points (*p* < 0.05, Duncan’s multiple range test).

To further investigate the potential role of the *SlDUF789* genes in responses to abiotic stress, this study analyzed its expression patterns under NaCl, PEG, and low-light treatments (Figure 9). Under NaCl treatment, *SlDUF789-7* exhibited a strong response to salt stress; its expression continued to rise after treatment, peaking at 24 h at 5.90 times that of the control, and then declined slightly at 48 h. *SlDUF789-10* and *SlDUF789-11* reached their peaks at 12 h, with expression levels 5.33-fold and 4.48-fold higher than the control, respectively. *SlDUF789-8* was continuously induced from 6 to 24 h, with expression levels remaining relatively high throughout the treatment period. *SlDUF789-5* and *SlDUF789-6* reached their highest expression levels at 24 and 48 h, respectively, at 4.52-fold and 3.89-fold that of the control. In contrast, *SlDUF789-4* showed only a brief induction at 6 h, with expression levels remaining below the control at all other time points; *SlDUF789-1* and *SlDUF789-2* peaked at 12 h, with expression levels 1.7-fold and 2.2-fold higher than the control, respectively.

Under PEG treatment simulating drought stress, *SlDUF789-8*, *-10*, and *-11* showed significant induction, reaching peak expression levels at 24 h, with expression levels 6.86, 6.24, and 5.46 times higher than the control, respectively; among them, *SlDUF789-11* maintained a relatively high expression level even at 48 h. *SlDUF789-7* responded rapidly, reaching its highest expression level at 6 h, which was 4.91 times that of the control. *SlDUF789-2* showed a brief upregulation at 6 h, while *SlDUF789-4* and *SlDUF789-6* peaked at 24 h. The expression levels of *SlDUF789-1* remained lower than those of the control group throughout the PEG treatment period.

Under low-light conditions, the expression of multiple *SlDUF789* genes was strongly induced. *SlDUF789-7*, *-9*, and *-11* exhibited the strongest responses to low-light; *SlDUF789-7* and *SlDUF789-9* peaked at 24 h, while *SlDUF789-11* peaked at 48 h, with expression levels 6.97-fold, 5.59-fold, and 8.48-fold higher than the control, respectively. Notably, *SlDUF789-11* exhibited sustained induction, with expression levels gradually increasing over time, suggesting that it may be involved in the sustained response to low-light stress. *SlDUF789-10* reached its highest expression level at 12 h, which was 4.22 times that of the control. *SlDUF789-5*, *-6*, and *-8* reached their peak expression at 24 h. In contrast, the overall expression level of *SlDUF789-1* remained low during the low-light treatment, while *SlDUF789-3* and *SlDUF789-4* showed significant suppression, with their expression levels consistently lower than those of the control group.

## 3. Discussion

DUF789 is a family of protein domains unique to plants; the functions of most of its members remain unclear, although existing evidence suggests that members of this family are involved in plant growth, development, and stress adaptation [1]. Although genome-wide identification of the DUF789 gene family has been conducted in species such as *Arabidopsis*, cotton, and soybean [25,26,27], no systematic analysis of this family in tomato has been reported to date. In this study, we identified 11 *SlDUF789* members in the tomato genome. These genes are unevenly distributed on seven chromosomes (Figure 1). This number is consistent with that of the diploid model plant *Arabidopsis thaliana* (11) [25], but lower than that of cotton (91 across four cotton species) and soybean (20) [26,27]. This difference in number is closely related to the species ploidy level: both tomato and Arabidopsis are diploid, with relatively stable family sizes; in contrast, the allopolyploid cotton has undergone significant family expansion through segmental and tandem duplications [26]; although soybean is a polyploid, its number of family members (20) is far lower than that of cotton, suggesting that, in addition to polyploidization, species-specific mechanisms of repeat retention or loss may be involved in phylogenetic divergence. Furthermore, *SlDUF789-2*, *-3*, and *-4* are densely arranged in a tandem configuration on chromosome 1. This pattern of local amplification is similar to the tandem duplication events observed in cotton [26] and may be evolutionarily or functionally related, with these genes synergistically participating in specific biological processes [28,29].

In terms of physicochemical properties, the length of tomato DUF789 proteins ranges from 87 to 1097 amino acids, which is significantly longer than that of *Arabidopsis* (186–409 aa) [25] but similar to that of soybean (144–1185 aa) and cotton (307–1203 aa) [26,27]. Compared to the narrow length distribution observed in *Arabidopsis*, events such as N- or C-terminal sequence extensions and the insertion or deletion of internal domain repeat units within this family may be more active in tomato. Notably, SlDUF789-6 (approximately 123.15 kDa) has a molecular weight exceeding 100 kDa; this phenomenon is similar to the extra-large DUF789 protein variants reported in cotton and may result from exon rearrangement, domain recruitment, or fusion with other functional motifs [26]. This suggests that the species-specific diversification of the DUF789 family at the protein structural level may have evolved relatively independently of changes in gene copy number. Most SlDUF789 proteins are acidic (pI < 7.0), a pattern similar to soybean GmDUF789 [27], which are mostly acidic with a few basic members. The GRAVY values of all SlDUF789 proteins are negative, a pattern identical to AtDUF789 and *GhDUF789* [25,26], supporting a conserved hydrophilic nature of the DUF789 family. Unlike all AtDUF789 members, which are uniformly unstable (II>40) [25], only SlDUF789-5 through SlDUF789-11 exceeded this threshold, with SlDUF789-1 to SlDUF789-4 predicted stable, a pattern comparable to stress-responsive GhDUF789 members [26]. Additionally, the aliphatic indices of SlDUF789 proteins are generally high (e.g.,>80), suggesting potential resilience to thermal denaturation despite their instability. Subcellular localization predictions indicate that SlDUF789 proteins are primarily localized to the nucleus (Table 1), with a small number of members localized to the plasma membrane, cell wall, chloroplasts, or mitochondria, suggesting that this family may perform dual functions involving both nuclear regulation and membrane/cell wall-related processes. Gene Ontology (GO) enrichment analysis of cotton GhDUF789 also supports its involvement in vesicular and membrane transport processes [26], which is consistent with the subcellular localization pattern of SlDUF789, suggesting that it may participate in cell wall metabolism or signal transduction via membrane transport pathways.

To investigate the evolutionary relationships within the *SlDUF789* family, this study utilized DUF789 protein sequences from tomato, *Arabidopsis*, soybean, and potato to construct a multispecies phylogenetic tree using the maximum likelihood method. The 11 tomato SlDUF789 proteins were resolved into five distinct phylogenetic subgroups; genes within the same subclade are likely to share similar functions (Figure 4). Analysis of conserved motifs and gene structure revealed that phylogenetically closely related members typically share similar motif arrangements and exon–intron structures (Figure 3), consistent with observations in the cotton *GhDUF789* and soybean *GmDUF789* families [26,27]. Notably, *SlDUF789-3* and *SlDUF789-6*, which belong to different subclades, exhibit significant differences in motif composition and gene structure; this pattern of differentiation is consistent with their phylogenetic positions, which not only supports the validity of the subclade classification, but also suggests potential functional differentiation among subclades [30]. Similar structural heterogeneity has also been reported in the soybean *DUF789* and tobacco *DUF868* families [27,31]; these structural differences may reflect a certain degree of structural differentiation among members of different subclades during evolution and provide clues regarding their potential functional differentiation. Gene duplication is a major driver of plant gene family expansion [30]. In this study, *SlDUF789-8* and *SlDUF789-10* were the only colinear gene pair detected in the tomato DUF789 family, suggesting minimal recent duplication with family size likely shaped by lineage-specific gene loss following the Solanaceae whole-genome triplication (WGT1) [28]. Their *Ka/Ks* ratio was less than 1 (Figure 5), reflecting strong purifying selection and functional constraint on the core DUF789 domain, which is consistent with the evolutionary characteristics of duplicated gene pairs in the cotton and soybean DUF789 families [26,27]. This contrast in coding-sequence conservation versus structural diversification among tomato members suggests that the DUF789 family maintains a conserved core function while accommodating species-specific structural divergence [30]. Furthermore, an interspecific collinearity analysis between tomato and *Arabidopsis* identified nine pairs of DUF789 homologs (Figure 5C); this cross-species collinearity is also consistent with reports in cotton and soybean [26,27], further supporting the functional conservation of this family across dicotyledons [30].

Gene expression patterns often provide important clues to their potential biological functions. Previous studies on the DUF789 family have shown that members of this family generally exhibit tissue- or developmental stage-specific expression in different plants, but their sites of high expression and potential functions suggest certain species-specific differences. For example, some members of the cotton *GhDUF789* family are consistently upregulated during fiber elongation and secondary wall formation, suggesting a possible involvement in fiber development and cell wall deposition processes [26]; in contrast, members of the soybean *GmDUF789* family exhibit differential expression in various tissues, including roots, stems, leaves, flowers, and seeds, indicating that these family members may have distinct expression regulatory patterns during the development of different organs [27]. Consistent with these studies, the present study found that *SlDUF789* family members also exhibit distinct expression patterns in different tomato tissues (Figure 7), suggesting that tissue- or developmental stage-specific expression may be one of the relatively conserved expression characteristics of *DUF789* family members. However, unlike cotton *GhDUF789* members, which are primarily expressed during fiber development, and soybean *GmDUF789* members, which show differential expression in multiple vegetative and reproductive organs [26,27], the high expression of tomato *SlDUF789* members is more concentrated in flower organs and stages related to fruit development. Specifically, *SlDUF789-6*, *-8*, and *-10* are highly expressed in fully open flowers; *SlDUF789-11* is highly expressed in unopened flower buds and mature-green fruits; whereas *SlDUF789-7* maintains high expression across multiple tissues and is particularly prominent in fully open flowers and red-ripe fruits (Figure 7). These results suggest that the tomato *SlDUF789* family may have developed expression patterns associated with tomato reproductive development and fleshy fruit development while retaining the tissue-specific expression characteristics of the DUF789 family. Furthermore, similar member-specific expression patterns have been observed in other DUF families, such as tomato DUF668 and tobacco DUF868 [3,31]. Although the types of domains contained in different DUF families vary, the differential expression of their members across different tissues or developmental stages suggests that the differentiation of expression regulation may be one of the key manifestations of functional diversification in plant DUF protein families. It is worth noting that the establishment of tissue-specific expression patterns is often co-regulated by *cis*-acting elements in the promoter and external signals. Therefore, further analysis of the composition of *SlDUF789* promoter elements and its dynamic expression patterns under hormonal and abiotic stress conditions will contribute to a more comprehensive understanding of the potential regulatory basis for the expression differentiation of this family’s members.

*Cis*-acting elements are an important component of regulatory networks governing plant hormone and stress responses; their compositional characteristics can provide crucial clues for elucidating gene transcriptional regulation patterns and potential functions [32]. In this study, multiple light-, hormone-, and stress-response-related *cis*-acting elements were identified in the promoter region of the *SlDUF789* gene (Figure 6). Among these, the TGACG and CGTCA motifs (methyl jasmonate response), TCA (salicylic acid response), ABRE (abscisic acid response), ARE (anaerobic induction), G-box, and box-4 elements were particularly abundant. ABRE is a core element in ABA-mediated responses to drought and osmotic stress [33]; hormone-responsive motifs such as TCA and TGACG/CGTCA are also widely distributed in the soybean *GmDUF789* promoter and are closely associated with adaptation to abiotic stress [27]; G-box and box-4 are frequently found together in the promoters of stress-responsive genes and participate in the cross-regulation between light signals and stress [34]. Although these predictions do not always correspond to actual transcriptional responses, differences in the composition of *cis*-acting elements among the promoters of different members may provide a potential transcriptional regulatory basis for their distinct hormonal and environmental responses.

To further analyze the consistency between promoter element prediction results and gene transcriptional responses, this study systematically analyzed the dynamic expression patterns of *SlDUF789* family members under ABA, MeJA, SA, NaCl, PEG, and low-light stress (Figure 8 and Figure 9). *SlDUF789-7* was significantly induced under ABA and salt stress, peaking at 24 h before declining slightly; this trend bears some similarity to the upregulation of soybean *GmDUF789* under salt and drought conditions [27]. Furthermore, members of the rice OsDUF668 and cotton GhDUF668 families also exhibited distinct expression responses under various abiotic stresses [35,36]. *SlDUF789-8* exhibited significant transcriptional activation under MeJA and PEG treatments, which is consistent with the expression patterns of soybean DUF family genes regulated by hormones and abiotic stresses in previous studies [27]; *SlDUF789-10* showed varying degrees of induction by MeJA, NaCl, and PEG, indicating that some family members can mount transcriptional responses to multiple hormones and environmental signals [34]. Notably, both *SlDUF789-7* and *SlDUF789-11* exhibited strong induction under low-light stress, which may be related to the abundance of elements such as G-box and box-4 in their promoter regions. G-box and box-4 are typical elements in photoreceptor signaling pathways and often co-occur with stress-related elements [34]. The cotton *GhDUF789* promoter is also rich in such elements, and some family members are significantly upregulated under drought and salt stress [26], suggesting that light and stress responses may be functionally coupled in the regulation of the DUF789 family. In contrast, *SlDUF789-1*, *-2*, *-3, -4*, and *-5* showed only mild induction or were repressed under most treatments, while *SlDUF789-9* exhibited strong induction primarily under low-light conditions, suggesting that members of the *SlDUF789* family exhibit distinct expression patterns under different hormonal and stress signals, reflecting functional differentiation among paralogous genes [37]. However, increased transcript levels under stress treatments do not necessarily indicate a direct functional role in plant resistance; such induction may reflect active participation in stress adaptation, regulatory feedback, or a secondary response to cellular perturbation [33]. Previous expression analyses of the DUF789 family in *Arabidopsis*, soybean, and cotton did not include low-light stress treatments [25,26,27]; although light-responsive elements were identified in cotton, changes in expression under low-light conditions were not further validated [26]. This means that the role of the DUF789 family in light signaling pathways has remained at the “predictive” level, lacking direct evidence of expression. Based on this, we hypothesize that the enrichment of elements including G-box and box-4 in the *SlDUF789* promoter is not coincidental but rather the structural basis for this family’s involvement in light signal transduction. This will provide clear candidate targets for subsequent validation of its role in low-light tolerance and stress resistance improvement through methods such as gene editing.

## 4. Materials and Methods

### 4.1. Whole-Genome Characterization and Analysis of Basic Traits of Tomato SlDUF789 Proteins

The tomato whole-genome data (version SL4.0) and corresponding annotation files were downloaded from the Sol Genomics Network database [https://solgenomics.net/ (accessed on 1 March 2026)]. The hidden Markov model (HMM) parameter file for the *DUF789* domain (PF05623) was obtained from the Pfam database [http://pfam.xfam.org/ (accessed on 1 March 2026)]. HMMER 3.0 software was used to perform homology searches on the complete tomato protein sequences, with an E-value threshold set at 1 × 10^−10^ [38]. The candidate sequences identified in the initial screening were further validated for conserved domains using the online tools SMART [http://smart.embl.de/ (accessed on 3 March 2026)] and NCBI CDD [https://www.ncbi.nlm.nih.gov/cdd/ (accessed on 4 March 2026)], and only sequences containing the complete *DUF789* domain were retained. We used the ExPASy-ProtParam tool to predict the physicochemical properties of SlDUF789 proteins [https://web.expasy.org/protparam/ (accessed on 6 March 2026)], including parameters such as molecular weight (kDa), isoelectric point (pI), and hydrophilicity (GRAVY) [39]. Plant-mPLoc v2.0 was used to predict the subcellular localization of these proteins [http://www.csbio.sjtu.edu.cn/bioinf/plant-multi/ (accessed on 7 March 2026)].

### 4.2. Chromosomal Mapping, Conservation of Motifs, and Gene Structure Analysis of Tomato SlDUF789 Genes

The “Gene Location Visualization” function in TBtools software (v2.0) was used to analyze the chromosomal distribution of tomato DUF789 family members, and the *SlDUF789* genes were systematically named based on their chromosomal locations. The MEME online tool [https://meme-suite.org/meme/ (accessed on 9 March 2026)] was employed to identify conserved motifs in the SlDUF789 protein sequences, and the results were visualized using TBtools software. Furthermore, the “Gene Structure Viewer” function in TBtools software was used to display the exon–intron structures of each *SlDUF789* family member [40].

### 4.3. Prediction of the Secondary and Tertiary Structures of Tomato SlDUF789 Proteins

Secondary structures of the SlDUF789 proteins, including α-helix, extended strand, β-turn, and random coil contents, were predicted using SOPMA [https://npsa-prabi.ibcp.fr/ (accessed on 11 March 2026)] [41]. Homology modeling via SWISS-MODEL was performed to obtain the tertiary structures of these proteins [https://swissmodel.expasy.org/ (accessed on 15 March 2026)] [42].

### 4.4. Phylogenetic Tree and Collinearity Analysis of the SlDUF789 Gene Family

The DUF789 protein sequences for tomato, *Arabidopsis*, soybean, and potato were downloaded from the Ensembl Plants database and the Sol Genomics Network (SGN) website, respectively. All sequences were combined into a single file, and a rootless phylogenetic tree was constructed using the maximum likelihood method in MEGA11 software (v11.0.11), with the bootstrap repetition set to 1000 [43] and all other parameters set to their default values. The phylogenetic tree was then optimized and visualized using the Evolview website [https://www.evolgenius.info/evolview/#/login (accessed on 14 March 2026)]. The tomato whole-genome sequence was aligned using TBtools software, and both intraspecific and interspecific collinearity analyses were performed using its built-in “One Step MCScanX Super Fast” feature.

### 4.5. Analysis of Promoter Cis-Acting Elements and Ka (Non-Synonymous)/Ks (Synonymous) Ratios of SlDUF789 Genes

The promoter sequences spanning 2000 bp upstream of the start codons of the tomato *SlDUF789* genes were extracted. *Cis*-acting elements in these sequences were identified using the PlantCARE online tool [https://bioinformatics.psb.ugent.be/webtools/plantcare/html/ (accessed on 19 March 2026)], and the results were visualized using TBtools software [44]. Based on the coding sequences (CDS) of the *SlDUF789* genes and their corresponding protein sequences, the “Simple *Ka/Ks* Calculator (NG)” feature built into TBtools was used to calculate the ratio of nonsynonymous substitution rate (Ka) to synonymous substitution rate (Ks) (*Ka/Ks*). This ratio was used to assess the selective pressures acting on the tomato *SlDUF789* gene family and its evolutionary dynamics [45].

### 4.6. Plant Materials and Treatment Methods

In this study, tomato (*S. lycopersicum* L. cv. Micro-Tom) was used as the experimental material. Seeds were surface-sterilized with 75% ethanol (Tianjin Fangdatongzheng Chemical Co., Ltd., Tianjin, China) and 10% sodium hypochlorite (Tianjin Beichen Fangzheng Reagent Factory, Tianjin, China), rinsed thoroughly with sterile distilled water, and soaked for 3–4 h to promote imbibition, followed by germination in the dark at 28 °C. Seedlings with emerged radicles were sown in a peat moss:vermiculite:perlite mixture (3:1:1, *v*/*v*/*v*). After the first true leaves unfolded, seedlings were transferred to a growth chamber (26/20 °C day/night, 250 μmol·m^−2^·s^−1^ light intensity, 16/8 h photoperiod) and grown hydroponically, initially in half-strength Hoagland solution for 2 d and then in full-strength Hoagland solution. At the three-leaf and one-heart stage, uniform plants were selected and exposed to stress treatments: 100 μM abscisic acid (ABA), 100 μM methyl jasmonate (MeJA), 100 μM salicylic acid (SA), 150 mM NaCl, 20% (*w*/*v*) PEG-6000 (Shanghai Yuanye Bio-Technology Co., Ltd., Shanghai, China), or low light (25 μmol·m^−2^·s^−1^). Leaf samples were taken at 0, 6, 12, 24, and 48 h post-treatment for qRT-PCR analysis [46].

For tissue-specific expression profiling analysis, roots and leaves were collected from untreated 21-day-old seedlings; unopened flower buds and fully opened flowers were harvested at 47 and 55 days after sowing, respectively; fruits were collected at 25 (mature green fruit) and 45 days (red-ripe fruit) after pollination. All samples were immediately frozen in liquid nitrogen and stored at −80 °C [47].

### 4.7. RNA Extraction and qRT-PCR Fluorescence Quantification

Total RNA was extracted from 0.5 g of tomato leaves using TRIzol reagent (Invitrogen, Carlsbad, CA, USA) [48]. The reverse transcription reaction was performed in a 10 μL reaction mixture containing 2 μL of 5× Evo M-MLV RT Master Mix (Accurate Biotechnology (Hunan) Co., Ltd., Changsha, China), 2 μL of RNA, and 6 μL of RNase-free water [49]. qRT-PCR reactions were performed in a 20 μL volume, consisting of 2 μL of cDNA, 0.4 μL of each primer, 7.2 μL of RNase-free water, and 10 μL of 2× SYBR Green Pro Taq HS Mix (Takara Biomedical Technology (Beijing) Co., Ltd., Beijing, China). Primers were designed using Primer 5.0 software. The amplification efficiency of each primer pair was determined by a standard curve generated from a 10-fold serial dilution of cDNA. Only primer pairs with efficiencies ranging from 90% to 110% were used in this study. qRT-PCR was performed on a LightCycler^®^ 96 real-time PCR system (Roche, Basel, Switzerland). The PCR cycling conditions were as follows: an initial pre-denaturation step at 95 °C for 30 s, followed by 40 cycles, each consisting of a 5 s incubation at 95 °C and a 30 s incubation at 60 °C. Each experiment was repeated three times (biological replicates), and gene expression levels were calculated using the 2^^-ΔΔCt^ method, i.e., the average of the replicate samples [50]. The sequences of the PCR amplification primers used in this study are detailed in Table 3.

### 4.8. Statistical Analysis

Statistical analyses were performed using SPSS 22.0 software (SPSS Inc., Chicago, IL, USA); all values are expressed as mean ± SE (*n* = 3 independent biological replicates). Statistical significance was determined using one-way ANOVA followed by Duncan’s multiple range test for multiple comparisons, with a significance level set at *p* < 0.05 [51]. GraphPad Prism 10.1.2 software was used to prepare the figures and tables (GraphPad Software, San Diego, CA, USA).

## 5. Conclusions

In this study, we systematically identified 11 members of the *SlDUF789* gene family in tomato and revealed their diversity in chromosomal distribution, protein properties, gene structures, conserved motifs, selection pressure, and phylogenetic relationships. Through collinearity and promoter analyses, we further clarified the evolutionary conservation and regulatory features of this family. Tissue expression analysis showed that *SlDUF789-6*, *-8*, *-10*, and *-11* were preferentially expressed in reproductive tissues, whereas *SlDUF789-7* was broadly expressed across all tested tissues, with relatively high expression in fully open flowers and red-ripe fruits. Hormone (ABA, MeJA, SA) and abiotic stress (NaCl, PEG, low light) treatments further revealed that *SlDUF789-7* exhibited significant expression changes under all tested conditions, whereas *SlDUF789-11* showed sustained upregulation under low-light stress, reaching the peak at 48 h. These results suggest that *SlDUF789* family members display diverse structures and distinct expression patterns in tomato, providing a valuable reference for further studies on the functions of this family in tomato development and stress responses.

## Figures and Tables

**Table 1:** Physicochemical properties predicted for SlDUF789.

Gene	Gene ID	Chr. No.	Chr. Location	Length (aa)	Mol. Wt. (Da)	pI	Instability Index	Aliphatic Index	Grand Average of Hydropathicity	Subcellular Localization
*SlDUF789-1*	*Solyc01g006080*	1	756,619–759,819	267	30,704.69	6.06	32.3	81.09	−0.379	Cell membrane. Chloroplast.
*SlDUF789-2*	*Solyc01g016510*	1	20,484,163–20,485,380	108	12,428.94	4.69	37.94	81.2	−0.336	Cell membrane. Cell wall.
*SlDUF789-3*	*Solyc01g016530*	1	20,488,064–20,488,775	87	10,029.02	4.46	32.79	57.13	−0.53	Nucleus.
*SlDUF789-4*	*Solyc01g016540*	1	20,490,180–20,490,832	138	15,737.64	5.84	35.15	72.9	−0.365	Cell membrane. Cell wall. Mitochondrion. Nucleus.
*SlDUF789-5*	*Solyc01g065560*	1	64,660,915–64,665,045	391	43,673.83	6.72	45.17	67.16	−0.573	Nucleus.
*SlDUF789-6*	*Solyc03g098630*	3	55,443,960–55,451,165	1097	123,147.52	8.42	52.79	71.09	−0.657	Nucleus.
*SlDUF789-7*	*Solyc05g008920*	5	3,170,849–3,177,750	408	45,253.56	5.61	51.54	68.19	−0.435	Nucleus.
*SlDUF789-8*	*Solyc07g054270*	7	62,530,600–62,533,134	309	35,824.05	4.91	65.33	64.98	−0.544	Cell wall. Chloroplast. Cytoplasm. Extracellular. Nucleus. Peroxisome.
*SlDUF789-9*	*Solyc09g075500*	9	63,429,549–63,435,548	432	48,400.29	8.7	59.99	74.93	−0.538	Nucleus.
*SlDUF789-10*	*Solyc10g008480*	10	2,476,260–2,479,325	315	36,560.24	4.89	52.65	70.57	−0.458	Chloroplast. Nucleus.
*SlDUF789-11*	*Solyc12g010730*	12	3,683,141–3,686,092	314	35,913.13	4.94	59.29	73.89	−0.49	Nucleus.

Note: pI, isoelectric point; Mol. wt., molecular weight. All values are predicted parameters, not experimentally measured.

**Table 2:** Predicted secondary structures of the SlDUF789 proteins.

Protein	Alpha Helix (%)	Extended Strand (%)	Beta Turn (%)	Random Coil (%)	Distribution of Secondary Structure Elements
SlDUF789-1	28.09	10.86	0	61.05	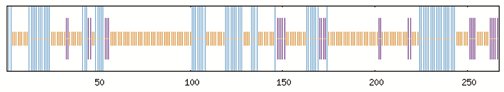
SlDUF789-2	25	11.11	0	63.89	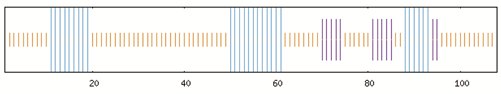
SlDUF789-3	16.09	12.64	0	71.26	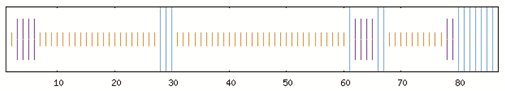
SlDUF789-4	27.54	10.87	0	61.59	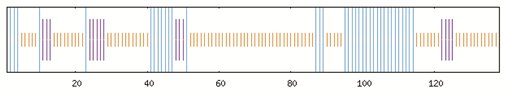
SlDUF789-5	22.76	7.16	0	70.08	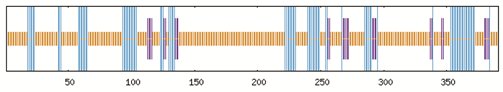
SlDUF789-6	12.76	6.02	0	81.22	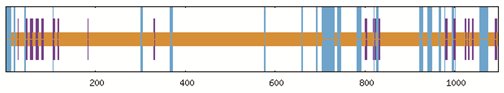
SlDUF789-7	22.79	7.6	0	69.61	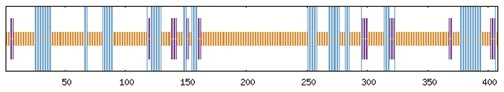
SlDUF789-8	30.10	8.41	0	61.49	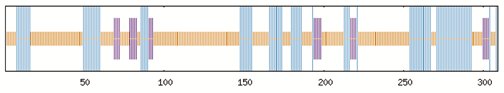
SlDUF789-9	21.53	8.33	0	70.14	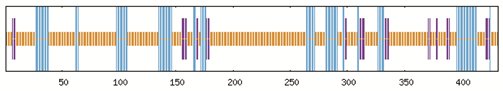
SlDUF789-10	26.03	7.62	0	66.35	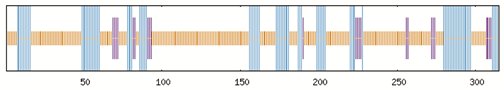
SlDUF789-11	24.52	11.46	0	64.01	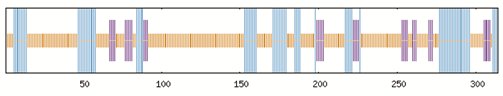

Note: Blue color indicates α-helices; purple color indicates extended chains; orange color indicates random coils.

**Table 3:** Primers for qRT-PCR.

Gene Name	Forward Primer (5′–3′)	Reverse Primer (5′–3′)
*SlActin*	AATGAACTTCGTGTGGCTCCAGAG	ATGGCAGGGGTGTTGAAGG
*SlDUF789-1*	TCTCCAGCAAGTTGGATGGC	TGTTGGCACCCTTTTTCTCA
*SlDUF789-2*	TCTTCAATCAGTCACACCGGT	GGAGCACCAACACCATATGC
*SlDUF789-3*	CGGAGTCTTGGAGTGATGCA	CCAAGGCGATCTCTTGAAGC
*SlDUF789-4*	TGCTGCAGATTCATGGCTGA	GCTCCAAGTTAGAATGCGCG
*SlDUF789-5*	AGAATTGAAGCGGGGAGTCG	TGGGAAAAACTTGAGCGGGA
*SlDUF789-6*	TTGCGTGCTGCTTTCTTGAC	CACAGTGCCTTGGCTGAAAC
*SlDUF789-7*	GCTGCCGTCGATGAATCAAC	CCACATCACACGTCCTCCAA
*SlDUF789-8*	GGCTATGGCATCCATGGGAA	CCAGCACCATAAGCACTCCA
*SlDUF789-9*	GCCCAGCATTTCTCTGAGGT	AGCTTCCCAAAGATCGCCAA
*SlDUF789-10*	CCAGCACTTCACCTGCAAAC	CTCAGAAGAGCATCCGTCCC
*SlDUF789-11*	GACTGAGTCTGCGTGGGAAA	CCCCCATCCGATCTCGAAAG

## Data Availability

The original contributions presented in this study are included in the article. Further inquiries can be directed to the corresponding author.
